# Humoral Response to SARS-CoV-2 Antigen in Patients Treated with Monoclonal Anti-CD20 Antibodies: It Is Not All about B Cell Recovery

**DOI:** 10.3390/neurolint14040075

**Published:** 2022-11-16

**Authors:** Julia Feige, Klaus Berek, Michael Seiberl, Patrick Hilpold, Wolfgang Hitzl, Franziska Di Pauli, Harald Hegen, Florian Deisenhammer, Eugen Trinka, Andrea Harrer, Peter Wipfler, Tobias Moser

**Affiliations:** 1 Department of Neurology, Christian Doppler University Hospital, Paracelsus Medical University and Center for Cognitive Neuroscience, European Reference Network EpiCARE, 5020 Salzburg, Austria; 2 Department of Neurology, Medical University of Innsbruck, 6020 Innsbruck, Austria; 3 Research Management (RM): Biostatistics and Publication of Clinical Studies Team, Paracelsus Medical University, 5020 Salzburg, Austria; 4 Department of Ophthalmology and Optometry, Paracelsus Medical University, 5020 Salzburg, Austria; 5 Research Program Experimental Ophthalmology and Glaucoma Research, Paracelsus Medical University, 5020 Salzburg, Austria; 6 Neuroscience Institute, Christian Doppler University Hospital, Paracelsus Medical University and Center for Cognitive Neuroscience, 5020 Salzburg, Austria; 7 Department of Dermatology and Allergology, Paracelsus Medical University, 5020 Salzburg, Austria

**Keywords:** COVID-19, antibody titers, B-cell depletion, B-cell repopulation, recall response, B-cell therapy, multiple sclerosis, vaccination, immunization

## Abstract

Anti-CD20 therapies decrease the humoral response to SARS-CoV-2 immunization. We aimed to determine the extent of the humoral response to SARS-CoV-2 antigens in correlation with peripheral B-cell dynamics among patients with central nervous system inflammatory disorders treated with anti-CD20 medications. We retrospectively included patients receiving anti-CD20 therapy after antigen contact who were divided into responders (>7 binding antibody units (BAU)/mL) and non-responders (<7 BAU/mL). In participants with first antigen contact prior to therapy, we investigated the recall response elicited once under treatment. We included 80 patients (responders *n* = 34, non-responders *n* = 37, recall cohort *n* = 9). The B-cell counts among responders were significantly higher compared to non-responders (mean 1012 cells/µL ± SD 105 vs. mean 17 cells/µL ± SD 47; *p* < 0.001). Despite very low B-cell counts (mean 9 cells/µL ± SD 20), humoral response was preserved among the recall cohort (mean 1653 BAU/mL ± SD 2250.1) and did not differ significantly from responders (mean 735 BAU/mL ± SD 1529.9; *p* = 0.14). Our data suggest that peripheral B cells are required to generate antibodies to neo-antigens but not for a recall response during anti-CD20 therapy. Evaluation of B-cell counts and pre-existing SARS-CoV-2 antibodies might serve as biomarkers for estimating the immune competence to mount a humoral response to SARS-CoV-2 antigens.

## 1. Introduction

B-cell depleting therapies are effective treatment options for many inflammatory diseases of the central nervous system (CNS) including multiple sclerosis (MS), neuromyelitis optica spectrum disorders (NMOSD) and autoimmune encephalitis (AE) [1]. Anti-CD20 therapeutics, such as rituximab and ocrelizumab, selectively target B cells and induce a profound, continuous depletion of peripheral B cells, based on a 6-monthly dosing interval [2,3]. Despite a mostly favorable safety profile, people undergoing anti-CD20 therapy are at risk of contracting severe infections and worse COVID-19 outcomes [4,5,6,7]. Susceptibility towards pathogens may not only be explained by B-cell depletion, but also relate to reduced humoral response to both infective agents and vaccines associated with anti-CD20 treatment [8,9,10]. While the exact mechanism behind inhibited antibody production associated with anti-CD20 medication remains elusive, it is clear that neutralizing antibodies have important roles in preventing and controlling infections. Biomarkers to predict efficient seroconversion following antigen contact in B-cell-depleted patients would therefore be highly appreciated. Personalized treatment intervals to allow B-cell recovery have been proposed as a rationale to optimize seroconversion rates, but extended dosing of anti CD20 therapies may diminish efficacy against the underlying inflammatory disease [11].

In the present study, we aimed to determine to what extent the humoral response to SARS-CoV-2 antigens depends on B-cell dynamics among patients with central nervous system inflammatory disorders on anti-CD20 therapy. 

## 2. Materials and Methods

### 2.1. Recruitment and Data Extraction

We conducted a retrospective observational analysis concerning patients with CNS inflammatory disorders treated with anti-CD20 therapies that have had contact with SARS-CoV-2, either by infection or by vaccination. Databases of both the University Hospitals of Salzburg and Innsbruck were searched. We only included patients for whom SARS-CoV-2-specific anti-spike IgG and correlating data on B-cell counts were available between December 2020 and May 2022. Anti-CD20 infusions (rituximab or ocrelizumab) were administered according to the summary of product characteristics (SmPC). Demographics, laboratory data and intervals of the last anti-CD20 infusion, antigen contact, B-cell assessment and anti-spike IgG level measurement were retrieved from electronic records. 

Depending on the time of the anti-CD20 therapy initiation, participants were divided into, first, two groups with established (treatment started prior to the first antigen contact) anti-CD20 therapy and secondly into the recall cohort, that consisted of patients who had had their first SARS-CoV-2 antigen contact prior to the start of anti-CD20.

Individuals with established anti-CD20 therapy were characterized as responders (>7 binding antibody units (BAU)/mL) and non-responders (<7 BAU/mL) according to their SARS-CoV-2 antibody levels. 

In order to ensure that the humoral response among the recall cohort was accurately elicited during anti-CD20 treatment and not a remnant from pre-treatment immunization, people in the recall cohort had to fulfill at least one of the following criteria:

- anti-spike IgG titers following recall SARS-CoV-2 contact increased compared to anti-CD20 pre-treatment levels;
- anti-CD20 therapy was ongoing for ≥12 months prior to the recall antigen exposure and to the respective anti-spike IgG assessment.

### 2.2. Laboratory Parameters

Assays were performed using standard laboratory methods at the Departments of Laboratory Medicine at the University Hospitals of Salzburg and Innsbruck. SARS-CoV-2-specific IgG antibody levels to the spike receptor-binding domain (RBD) were evaluated by the SARS-CoV-2 IgG II Quant assay (Abbott Laboratories) measured on the Architect I 2000 SR in accordance with the manufacturer’s instructions as previously described [12]. The detection limit of the antibody assay is 7 BAU/mL, which was used to categorize participants of this study into responders and non-responders. Correlating B-cell counts were obtained from routine diagnostic lymphocyte subset analysis in patients on anti-CD20 therapy. 

### 2.3. Statistics

Data were checked for consistency and normality. Data are presented as mean ± standard deviation (SD) if not otherwise specified. Fisher’s Exact test or Pearson’s Chi-square test were used to compare the categorical variables. ANOVA, Welch-ANOVA with least significant difference (LSD) tests were used to test normally distributed variables. In case of non-normality, generalized linear models with log normal distribution were used for continuous variables. Whisker plots were used to illustrate the 95% confidence intervals (CI) for means. All reported tests were two-sided, and *p*-values < 0.05 were considered statistically significant. All statistical analyses in this report were performed by use of STATISTICA 13 (Hill, T. and Lewicki, P.; *Statistics: Methods and Applications*. StatSoft, Tulsa, OK). The schematic figure was created by BioRender.com.

### 2.4. Ethics

This retrospective study was conducted in accordance with national regulations and legislative directives, in respect of anonymous, retrospective analysis of data derived from routine diagnostic procedures. Moreover, the study was covered by the approval of the local Ethics Committee (415-E/161 2111-2018) for the ongoing study on immunological processes in inflammatory CNS diseases. We adhered to principles of Good Clinical Practice (GCP) as defined by the International Conference on Harmonization (ICH), to the Declaration of Helsinki and the Austrian Data Safety Authority instructions.

## 3. Results

### 3.1. Demographics

We included 80 patients in this study. A total of 74 patients (93%) had been diagnosed with MS; 16 (20%) with a relapsing remitting disease course, 26 (33%) with a primary progressive and 32 (40%) with a secondary progressive disease course. Of the remaining six individuals (“others”), five patients had been diagnosed with NMOSD and one patient was considered to suffer from an inflammatory CNS disease with unknown etiology; however, a progressive disease course of MS was among the differentials. Mean age was 48 years ± SD 12. According to their antibody levels, 37 people were categorized as non-responders and 34 as responders. The recall cohort consisted of nine individuals. A minority of 12 patients (15%) had been diagnosed with COVID-19 in the past, and for 8 of them their last SARS-CoV-2 antigen contact prior to study inclusion was by infection. Among the remaining 68 (85%) individuals, the humoral response stemmed from vaccination. Demographics of the three groups are displayed in Table 1.

### 3.2. B Cell Recovery and Humoral Response

Peripheral B cells were fully depleted in 30/37 (81%) of non-responders, in 2/34 (6%) of responders and in 7/9 (78%) of the recall cohort. Time from last SARS-CoV-2 antigen contact to antibody assessment was shortest for the recall cohort and similar among the individuals with established anti-CD20 therapy (recall: mean 67 days ± SD 63.9, non-responders: mean 92 days ± SD 65.2, responders: mean 91 days ± SD 74.5, Table 1). Antibody and B-cell assessment were in mean 17 days (±SD 52) apart among the responders and 12 days (±SD 63) apart among the non-responders. For every individual among the recall cohort, antibody levels and B-cell counts were measured on the same day.

**Figure 1:**
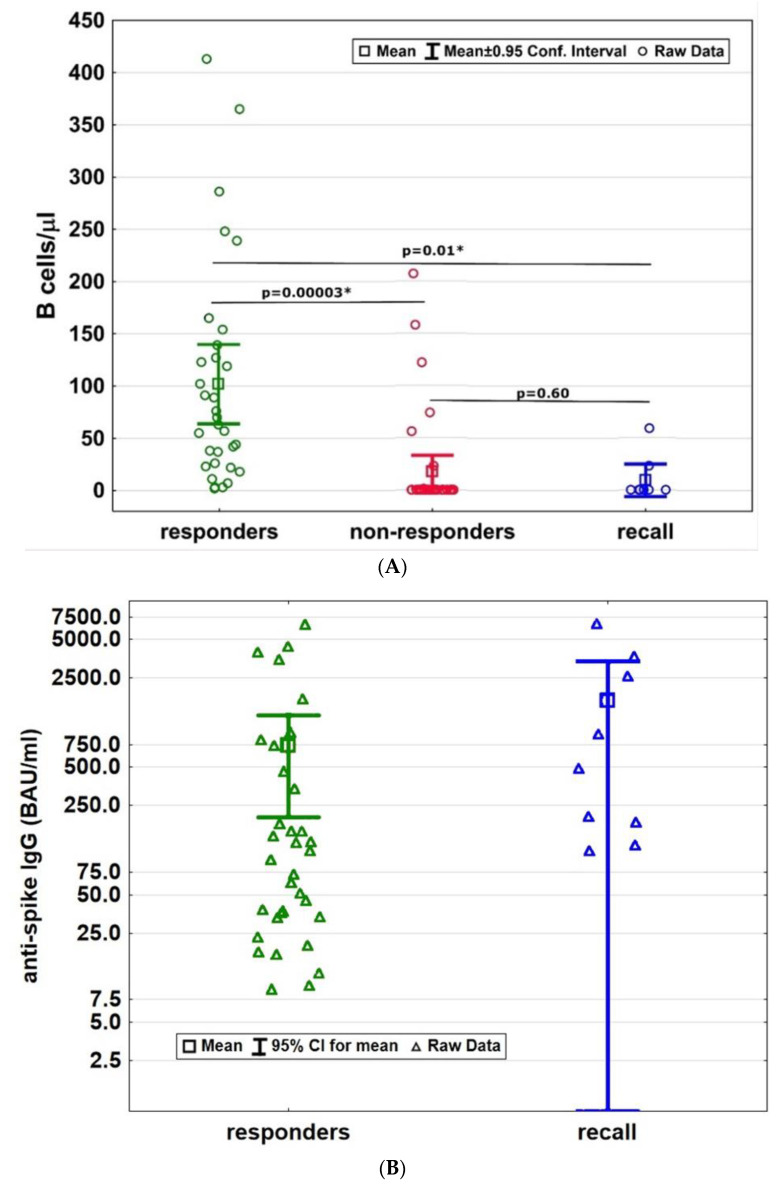

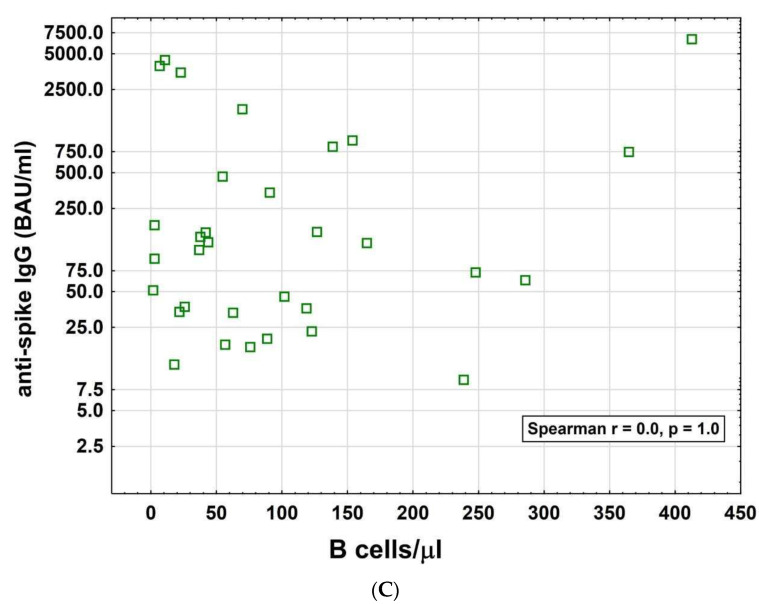
(**A**) Peripheral B-cell counts among all three patient groups at the time of anti-spike IgG assessment. B-cell numbers are highest in the responder cohort compared to the non-responders and the recall cohort. B cells among the non-responders and among the recall cohort were not statistically different. (**B**) Humoral response to SARS-CoV-2 antigens in terms of anti-spike IgG antibody levels were similar among the responder and recall cohort. (**C**) We found no correlation between B-cell counts and amount of anti-spike IgG levels among the responders. BAU = binding antibody units; CI = confidence interval; IgG = Immunoglobulin G; * = statistically significant.

Responders had significantly higher B-cell counts compared to non-responders (B-cell counts: responders: mean 102 cells/µL ± SD 105; non-responders: mean 17 cells/µL ± SD 47; *p* < 0.001, Figure 1A). The responder cohort had in mean 735 BAU/mL ± SD 1529.9 anti-spike IgG levels (Figure 1B). The amount of anti-spike IgG levels did not correlate with B-cell counts among the responders (*p* = 1.0, Figure 1C). 

All patients among the recall cohort had preserved humoral responses following recall antigen contact. The mean anti-spike IgG antibody levels of the recall cohort was 1653 BAU/mL (±SD 2250.1) and did not significantly differ from the responder cohort (*p* = 0.14, Figure 1B). B-cell counts (9 cells/µL ± SD 20) among the recall cohort were comparably low as in the non-responder group and significantly below B-cell numbers of the responder cohort (*p* = 0.01, Figure 1A). The amount of anti-spike IgG levels did not correlate with B-cell counts among the recall cohort (*p* = 0.13). A total of 88% among the recall cohort and 38% among the responders had B-cell counts below the recently proposed cut-off value of 40 cells/µL [13].

## 4. Discussion

The most intriguing finding from our report is that re-emergence of peripheral B cells is not a conditio sine qua non for the generation of a pathogen-specific antibody response. Namely, B cell repopulation does not appear to be necessary in patients with established humoral immunological memory to induce an adequate response to recall antigens. Among the recall cohort in our study, the B-cell counts were comparable to the non-responders group. Nevertheless, antibody production following recall antigen contact was adequate. However, the recall cohort consisted of only a relatively small cohort and the findings need to be confirmed in larger cohorts. Preserved humoral immunologic memory despite B-cell depletion has been reported earlier and appears to rely on the fact that terminally differentiated plasma cells, the main source of antibodies, are spared by anti-CD20 therapy [13,14]. Similar observations were made among patients with MS under cladribine treatment. This oral immune reconstitution therapy also induces profound B-cell depletion without impacting on pre-existing pathogen-specific antibody levels [15,16,17]. Unlike the effect of anti-CD20 medications, transient lymphodepletion by cladribine appears not to interfere with humoral responses to neo antigens [18].

We corroborated recent findings that among patients with established anti-CD20 therapy, the humoral response to COVID-19 antigens is associated with B-cell recovery dynamics [19,20]. However, according to our study, the amount of antibody levels does not seem to be related to the extent of peripheral B-cell counts. Therefore, our data suggest that if peripheral B-cell re-emergence is awaited to increase seroconversion rates, immunization can occur soon after the first B cells are detectable. The treatment interval should not unnecessarily be prolonged in order to avoid disease reactivation of the underlying inflammatory disorder. Our data are in contrast to a recent report suggesting that an optimal humoral response is only elicited once peripheral B cells have reached the range of 40 cells/µL [20]. Other authors have suggested a cut-off level of 1 B cell/µL as a predictive condition required for seroconversion [19]. Although extended dosing intervals appear to be associated with a low risk of disease reactivation in MS, a personalized treatment regimen to improve the humoral response may only be considered after careful weighing of the risk–benefit [21,22]. This appears especially important in the light of observations that the immunological long-term effects of anti-CD20 therapy might last for over 18 months after treatment discontinuation [12].

**Figure 2:**
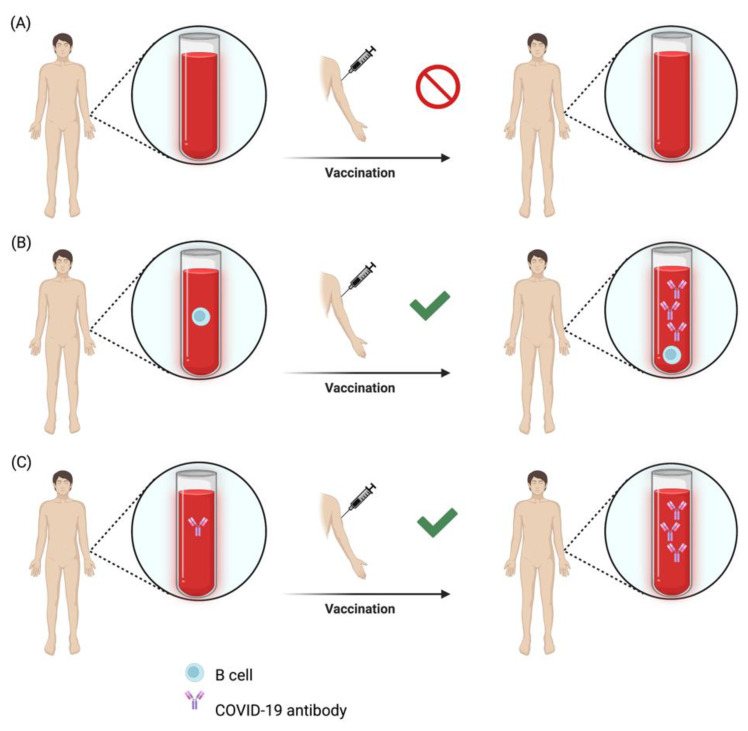
From our findings, three possible scenarios can be deduced to assess the probability of a successful humoral response to COVID-19 vaccination in people under anti-CD20 therapy. (**A**) In a B-cell-depleted person without detectable pathogen-specific antibodies, vaccination likely will not result in seroconversion. (**B**) Once peripheral B-cell repopulation has started, a humoral response to vaccines can be expected. (**C**) In individuals with existing pathogen-specific antibody levels, further antigen contact (recall response) can elicit an adequate antibody production independent of peripheral B-cell recovery. The schematic figure was created with BioRender.com (2022).

Our study suggests that both B-cell repopulation and existing B-cell memory might represent a useful marker to predict the humoral response to pathogen-specific antigen contact (Figure 2). To estimate the probability of seroconversion, we therefore suggest not only the assessment of B-cell counts but also the determination of whether pathogen-specific antibodies are detectable at the time of vaccine administration. Among individuals with pre-existing antibody titers, awaiting B-cell recovery may not only be unnecessary, but also associated with an increased risk regarding reactivation of the underlying inflammatory disorder. Our study strongly suggests COVID-19 booster shots to be effective even with concomitant B-cell depletion in individuals with a preformed pathogen-specific humoral immunologic memory. The implications for the management of patients under anti-CD20 therapy will provide valuable guidance for clinical decision-making. Our findings on preserved recall responses under anti-CD20 therapies can likely be expanded to other vaccines and may be an attractive feature for patients and treating physicians.

Nonetheless, these results must be interpreted with caution due to the following limitations. Importantly, the sample size of our recall cohort is small and recall responses in people with concomitant anti-CD20 therapy should be investigated in larger, prospective cohorts. As data were not collected prospectively, the timing of the anti-spike titer assessment was not harmonized. In particular, the study is limited by the fact that B-cell assessment was not undertaken at the time of antigen contact, but in temporal relationship with antibody assessment. Importantly, no patient had, however, received an anti-CD20 infusion during the period of last antigen contact and antibody assessment. Furthermore, this study has not considered the possible cumulative effect of repeated antigen contact although it is known that SARS-CoV-2 antibody levels clearly decrease with time [23]. Lastly, we considered that the detected number of B cells resulted from repopulation rather than from incomplete depletion.

## 5. Conclusions

Within the limits of the relatively small cohorts investigated, our data suggest that peripheral B cells are required to generate pathogen-specific antibodies to neo-antigens but not for recall response in patients treated with anti-CD20 therapy. We thus propose that B cell repopulation and pre-existing COVID-19 antibodies may both serve as biomarkers for efficient humoral responses to SARS-CoV-2 antigens. However, additional studies are needed to further address these issues.

## Figures and Tables

**Table 1:** Demographic features and patient characteristics among the three cohorts.

Characteristics	Responders (>7 BAU/mL)	Non-Responders (<7 BAU/mL)	Recall Cohort	*p*-Values
*n* (%)	34 (43)	37 (46)	9 (11)	n.s.
Diagnosis, n (%)				n.s.
● RRMS	7 (21)	7 (19)	2 (22)	
● PPMS	6 (18)	15 (41)	5 (56)	
● SPMS	20 (59)	11 (30)	1 (11)	
● Others	1 (3)	4 (11)	1 (11)	
Mean age (years, ±SD)	52 ± 12	46 ± 11	45 ± 12	0.04
Female, *n* (%)	16 (47)	24 (65)	3 (33)	n.s.
COVID-19 infection, n (%)	5 (15)	5 (14)	2 (22)	n.s.
Anti-CD20 agent, n (%)				n.s.
● ocrelizumab	12 (35)	24 (65)	6 (67)	
● rituximab	22 (65)	13 (35)	3 (33)	
Mean intervals between last infusion and B-cell count (months, ±SD)	17 ± 11.6	7 ± 4.3	6 ± 3.1	0.01
Mean intervals between last SARS-CoV-2 contact and antibody assessment (days, ±SD)	91 ± 74.5	92 ± 65.2	67 ± 63.9	n.s.
B cells/µL at the time of antibody assessment (mean, ±SD)	101.8 (±105.4)	17.3 (±47.4)	9.1 (±20.2)	0.01

*n* = number; F = female; M = male; SD = standard deviation; MS: multiple sclerosis; RRMS = relapsing remitting MS; PPMS = primary progressive MS; SPMS = secondary progressive MS; BAU = binding antibody units; n.s. = not significant.

## Data Availability

The data that support the findings of this study are available on reasonable request from the corresponding author.

## References

[1] Hohlfeld R. (2018). B-cells as therapeutic targets in neuro-inflammatory diseases. Clin. Immunol..

[2] Montalban X., Hauser S.L., Kappos L., Arnold D.L., Bar-Or A., Comi G., de Seze J., Giovannoni G., Hartung H.P., Hemmer B. (2017). Ocrelizumab versus Placebo in Primary Progressive Multiple Sclerosis. N. Engl. J. Med..

[3] Hauser S.L., Waubant E., Arnold D.L., Vollmer T., Antel J., Fox R.J., Bar-Or A., Panzara M., Sarkar N., Agarwal S. (2008). B-cell depletion with rituximab in relapsing-remitting multiple sclerosis. N. Engl. J. Med..

[4] Sormani M.P., De Rossi N., Schiavetti I., Carmisciano L., Cordioli C., Moiola L., Radaelli M., Immovilli P., Capobianco M., Trojano M. (2021). Disease-Modifying Therapies and Coronavirus Disease 2019 Severity in Multiple Sclerosis. Ann. Neurol..

[5] Louapre C., Maillart E., Papeix C., Zeidan S., Biotti D., Lepine Z., Wahab A., Zedet M., Labauge P., Tilikete C. (2021). Outcomes of coronavirus disease 2019 in patients with neuromyelitis optica and associated disorders. Eur. J. Neurol..

[6] Luna G., Alping P., Burman J., Fink K., Fogdell-Hahn A., Gunnarsson M., Hillert J., Langer-Gould A., Lycke J., Nilsson P. (2020). Infection Risks Among Patients With Multiple Sclerosis Treated With Fingolimod, Natalizumab, Rituximab, and Injectable Therapies. JAMA Neurol..

[7] Simpson-Yap S., De Brouwer E., Kalincik T., Rijke N., Hillert J.A., Walton C., Edan G., Moreau Y., Spelman T., Geys L. (2021). Associations of Disease-Modifying Therapies With COVID-19 Severity in Multiple Sclerosis. Neurology.

[8] Louapre C., Ibrahim M., Maillart E., Abdi B., Papeix C., Stankoff B., Dubessy A.L., Bensa-Koscher C., Creange A., Chamekh Z. (2022). Anti-CD20 therapies decrease humoral immune response to SARS-CoV-2 in patients with multiple sclerosis or neuromyelitis optica spectrum disorders. J. Neurol. Neurosurg. Psychiatry.

[9] Zabalza A., Cardenas-Robledo S., Tagliani P., Arrambide G., Otero-Romero S., Carbonell-Mirabent P., Rodriguez-Barranco M., Rodriguez-Acevedo B., Restrepo Vera J.L., Resina-Salles M. (2021). COVID-19 in multiple sclerosis patients: Susceptibility, severity risk factors and serological response. Eur. J. Neurol..

[10] Apostolidis S.A., Kakara M., Painter M.M., Goel R.R., Mathew D., Lenzi K., Rezk A., Patterson K.R., Espinoza D.A., Kadri J.C. (2021). Cellular and humoral immune responses following SARS-CoV-2 mRNA vaccination in patients with multiple sclerosis on anti-CD20 therapy. Nat. Med..

[11] Baker D., MacDougall A., Kang A.S., Schmierer K., Giovannoni G., Dobson R. (2022). Seroconversion following COVID-19 vaccination: Can we optimize protective response in CD20-treated individuals?. Clin. Exp. Immunol..

[12] Moser T., O’Sullivan C., Otto F., Hitzl W., Pilz G., Schwenker K., Mrazek C., Haschke-Becher E., Trinka E., Wipfler P. (2022). Long-term immunological consequences of anti-CD20 therapies on humoral responses to COVID-19 vaccines in multiple sclerosis: An observational study. Ther. Adv. Neurol. Disord..

[13] Moser T., Otto F., O’Sullivan C., Hitzl W., Pilz G., Harrer A., Trinka E., Wipfler P. (2022). Recall response to COVID-19 antigen is preserved in people with multiple sclerosis on anti-CD20 medications—A pilot study. Mult. Scler. Relat. Disord..

[14] Ziemssen T., Bar-Or A., Arnold D., Comi G., Hartung H.-P., Hauser S.L., Lublin F., Selmaj K., Traboulsee A., Chin P. (2017). P 2 Effect of ocrelizumab on humoral immunity markers in the phase iii, double-blind, double-dummy, IFNβ-1a–controlled OPERA I and OPERA II studies. Clin. Neurophysiol..

[15] Moser T., O’Sullivan C., Puttinger C., Feige J., Pilz G., Haschke-Becher E., Cadamuro J., Oberkofler H., Hitzl W., Harrer A. (2021). Pre-Existing Humoral Immunological Memory Is Retained in Patients with Multiple Sclerosis Receiving Cladribine Therapy. Biomedicines.

[16] Moser T., Schwenker K., Seiberl M., Feige J., Akgun K., Haschke-Becher E., Ziemssen T., Sellner J. (2020). Long-term peripheral immune cell profiling reveals further targets of oral cladribine in MS. Ann. Clin. Transl. Neurol..

[17] Baker D., MacDougall A., Kang A.S., Schmierer K., Giovannoni G., Dobson R. (2022). CD19 B cell repopulation after ocrelizumab, alemtuzumab and cladribine: Implications for SARS-CoV-2 vaccinations in multiple sclerosis. Mult. Scler. Relat. Disord..

[18] Achiron A., Mandel M., Dreyer-Alster S., Harari G., Magalashvili D., Sonis P., Dolev M., Menascu S., Flechter S., Falb R. (2021). Humoral immune response to COVID-19 mRNA vaccine in patients with multiple sclerosis treated with high-efficacy disease-modifying therapies. Ther. Adv. Neurol. Disord..

[19] Kornek B., Leutmezer F., Rommer P.S., Koblischke M., Schneider L., Haslacher H., Thalhammer R., Zimprich F., Zulehner G., Bsteh G. (2022). B Cell Depletion and SARS-CoV-2 Vaccine Responses in Neuroimmunologic Patients. Ann. Neurol..

[20] Tolf A., Wiberg A., Muller M., Nazir F.H., Pavlovic I., Lauren I., Mangsbo S., Burman J. (2022). Factors Associated With Serological Response to SARS-CoV-2 Vaccination in Patients With Multiple Sclerosis Treated With Rituximab. JAMA Netw. Open.

[21] Maarouf A., Rico A., Boutiere C., Perriguey M., Demortiere S., Pelletier J., Audoin B., Under the aegis of OFSEP (2020). Extending rituximab dosing intervals in patients with MS during the COVID-19 pandemic and beyond?. Neurol. Neuroimmunol. Neuroinflamm.

[22] Rolfes L., Pawlitzki M., Pfeuffer S., Nelke C., Lux A., Pul R., Kleinschnitz C., Kleinschnitz K., Rogall R., Pape K. (2021). Ocrelizumab Extended Interval Dosing in Multiple Sclerosis in Times of COVID-19. Neurol. Neuroimmunol. Neuroinflamm.

[23] Ibarrondo F.J., Hofmann C., Fulcher J.A., Goodman-Meza D., Mu W., Hausner M.A., Ali A., Balamurugan A., Taus E., Elliott J. (2021). Primary, Recall, and Decay Kinetics of SARS-CoV-2 Vaccine Antibody Responses. ACS Nano.

